# Sulodexide for limb salvage in refractory diabetic foot ulcer with arteriosclerosis obliterans: a case report

**DOI:** 10.3389/fendo.2026.1756228

**Published:** 2026-02-12

**Authors:** Zhaoyan Chen, Wei Lin

**Affiliations:** Department of Orthopedics, People’s Hospital of Beiliu, Beiliu, China

**Keywords:** diabetic foot ulcer, limb salvage, microcirculation, peripheral arterial disease, sulodexide

## Abstract

**Background:**

Patients with diabetic foot ulcers (DFU) remain at substantial risk for impaired wound healing, even following successful revascularization. This clinical challenge is often associated with underlying microcirculatory dysfunction, highlighting a critical unmet need for effective intervention strategies.

**Case description:**

A 77-year-old female patient was suffering from severe DFU on her right foot, with infected wound, complicated by arteriosclerosis obliterans of the lower limbs. She underwent surgical debridement and angiographically and hemodynamically successful endovascular angioplasty. Despite these interventions and restored macrovascular perfusion, the wound exhibited continuously deterioration, and the patient was at imminent risk of minor amputation. Therefore, persistent microcirculatory dysfunction was considered a major contributing factor to the poor healing response, prompting the initiation of a salvage regimen with intravenous Sulodexide (600 LSU/day). Following a 51-day Sulodexide treatment, complete wound healing was achieved and the patient was discharged.

**Conclusion:**

This case report suggests that highly selected DFU patients unresponsive to standard therapies including successful revascularization, Sulodexide may be a plausible salvage or adjunctive therapeutic option. This observation is strictly hypothesis-generating and its potential role warrants investigation in future prospective clinical trials.

## Introduction

Diabetic foot ulcer (DFU) is one of the most severe and potentially fatal chronic complications of diabetes mellitus (DM), posing a significant issue in global public health (1). The pathophysiology of DFU is multifactorial, stemming primarily from chronic hyperglycemia. This process involves a confluence of key pathologies, including angiopathy, peripheral neuropathy, immune dysfunction, and infection. These pathologies act synergistically to impair local tissue viability and healing capacity, culminating in ischemia, tissue necrosis, and a profound susceptibility to infection (2). It is estimated that 19-34% of patients with DM will develop a DFU (1). Furthermore, peripheral arterial disease (PAD) is an independent risk factor for DFU that also severely compromises the healing process, thereby increasing the risk of infection and subsequent limb amputation (1, 3). The substantial disease burden and poor prognosis associated with DFU render its effective management a significant clinical challenge, underscoring the urgency for improved therapeutic strategies.

For patients with DFU and concomitant PAD, establishing adequate tissue perfusion via limb revascularization is a necessary condition for promoting wound healing. However, a subset of patients exhibit non-healing or deteriorating wounds following successful endovascular or bypass procedures, despite restored macrovascular perfusion (4). The pathophysiological basis for this phenomenon is persistent microcirculatory dysfunction, primarily centered on endothelial dysfunction, which fosters the formation of a local pro-inflammatory and pro-thrombotic state (5). This pathology at the microvascular level critically undermines the perfusion improvements conferred by revascularization, thus constituting a key determinant of therapeutic failure.

Therefore, targeting microcirculatory function is a promising therapeutic strategy for these refractory DFU. Sulodexide is a glycosaminoglycan mixture composed of 80% low-molecular-weight heparan sulfate and 20% dermatan sulfate. Its multifactorial pharmacological profile, encompassing vasoprotective, anti-thrombotic, and hemorheological properties, renders it a promising therapeutic candidate (6). However, despite a strong mechanistic basis, and previous studies indicating that sulodexide can improve microcirculatory perfusion in mild to moderate DFU, its clinical role in the treatment of severe DFU remains unclear, especially regarding its efficacy in complex situations following successful revascularization (7). Accordingly, this case report describes a patient with a DFU refractory to both standard therapy and revascularization who subsequently achieved limb salvage with Sulodexide. The purpose is to provide preliminary evidence for the potential efficacy of this drug in this specific clinical scenario.

## Case description

A 77-year-old woman was admitted to our center on February 13, 2025, presenting with a 12-day history of erythema, edema, and pain in the right foot, with associated gangrene of the second toe. The past medical history was notable for type 2 diabetes mellitus (T2DM) of over 10 years’ duration, characterized by suboptimal glycemic control with poor adherence to oral antidiabetic agents. Comorbid conditions included hypertension and a history of coronary heart disease, which was treated with percutaneous coronary intervention (PCI) and single tent implantation in 2013. The patient had no known family history of diabetes-related complications, her psychosocial history was unremarkable. On physical examination at admission, the right foot exhibited severe pitting edema with dusky erythema. Dry gangrene was present at the distal aspect of the second toe, and the third toe was cool to the touch with cyanotic discoloration. The dorsalis pedis and posterior tibial arterial pulses were absent bilaterally on palpation. Testing with a 10-g Semmes-Weinstein monofilament revealed a complete loss of protective sensation on the plantar surface. Initial laboratory findings were indicative of a significant systemic inflammatory response, with a white blood cell count of 15.84×10^9^/L and a high-sensitivity C-reactive protein level of 92.40 mg/L. The severity of the infection was further highlighted by a subsequent rise in hs-CRP to a peak of 134.3 mg/L on February 17, following the initial debridement. With continued antibiotic therapy and further surgical management, the hs-CRP level demonstrated a consistent downward trend, eventually normalizing to 5.0 mg/L by March 7. Computed tomography angiography (CTA) of the lower limb arteries confirmed severe PAD, revealing diffuse calcification of the arterial walls from the femoral to the ankle level, with multi-segment severe stenosis and occlusions. Specifically on the right, the distal anterior tibial and peroneal arteries were occluded, the posterior tibial artery was severely stenotic, and the plantar arch was poorly visualized. In conjunction with the clinical manifestations and laboratory findings, the diagnosis upon admission was established as a right diabetic foot ulcer with infection and toe gangrene, complicated by bilateral lower extremity arteriosclerosis obliterans. After admission, a systematic and multidisciplinary treatment plan was initiated. The detailed medication regimen is presented in **Table 1**.

**Table 1:** Main drug therapy regimen during the patient’s hospitalization.

Drug category	Drug name	Dosage & administration	Treatment duration
Microcirculation-improving drug	Sulodexide	600 LSU/dose, Qd, intravenous infusion	2025-3–7 to 2025-4-26
Anti-infective drug	Amoxicillin and clavulanate potassium	1.2 g/dose, Q8h, intravenous infusion	2025-2–13 to 2025-2-15
	Cefuroxime sodium	1.5 g/dose, Qd, intravenous infusion	2025-2-26
	Cefoperazone-sulbactam sodium	2 g/dose, Qd, intravenous infusion	2025-3–3 to 2025-3-18
Analgesic drug	Naproxen/codeine	330 mg/dose, Bid, oral	2025-2–13 to 2025-2-18
	Parecoxib sodium	40 mg/dose, Q12h, intravenous injection	2025-3–4 to 2025-3-6; 2025-4-7
Anticoagulant drug	Rivaroxaban	0.5 mg/dose, Qd, oral	2025-2–26 to 2025-3-22
Hypoglycaemic drug	Insulin aspart	1–5 IU/dose, Tid, subcutaneous injection (adjusted according to blood glucose dynamics)	2025-2–13 to 2025-4-25
Antihypertensive drug	Nifedipine sustained-release tablets	20 mg/dose, Bid, oral	2025-2–13 to 2025-4-25
Lipid-lowering drug	Atorvastatin calcium	20 mg/dose, Qd, oral	2025-2–26 to 2025-3-24
Antiplatelet drug	Indobufen	0.1 g/dose, Bid, oral	2025-2–26 to 2025-3-2
	Enteric-coated aspirin	100 mg/dose, Qd, oral	2025-4–21 to 2025-4-25

To prevent local extension of the infection, the patient underwent sharp debridement with amputation of the right second toe and corresponding metatarsal head on February 16, 2025. Postoperatively, the wound was managed with negative pressure wound therapy (NPWT) at a continuous pressure of -130 mmHg. Alongside local wound care, a multi-modal offloading strategy was strictly implemented throughout the entire treatment period. During hospitalization, this consisted of strict bed rest and the use of a removable offloading shoe whenever the patient was transferred. Upon discharge, the patient continued to use the offloading shoe and was provided with a wheelchair to ensure non-weight-bearing of the affected foot. The dressing change interval was dictated by the volume of wound effusion and clinical signs of infection. Subsequently, to address the underlying critical lower limb ischemia, percutaneous transluminal angioplasty (PTA) of the right lower limb artery was performed on February 24, 2025. This procedure involved successful balloon angioplasty of the severely stenotic posterior tibial artery. Macrovascular perfusion was successfully restored, as confirmed by both postoperative angiography showing vessel patency and a significant improvement in the ankle-brachial index (ABI) from a preoperative value of 0.54 to 0.92 postoperatively. However, this hemodynamically successful revascularization failed to translate into the expected clinical benefits at the tissue level. Within a few days post-procedure, the patient’s rest pain persisted, the affected foot remained cool, and the surgical wound showed no evidence of granulation tissue formation. This clinical observation was supported by transcutaneous oximetry (TcPO_2_), which showed minimal improvement from a preoperative value of 25.4 mmHg (February 18, 2025) to 28.6 mmHg post-revascularization (March 6, 2025).

Owing to persistent tissue ischemia, the clinical condition of the foot deteriorated rapidly, and the third toe progressed to established ischemic gangrene. To manage the advancing tissue necrosis and prevent further local extension of the infection, a second surgical debridement involving amputation of the third toe and metatarsal was performed on March 4, 2025. Postoperatively, the enlarged wound bed remained edematous and exhibited marked pallor, failing to demonstrate any signs of granulation tissue formation. Consequently, the limb was deemed unsalvageable, and minor amputation was advised as the definitive treatment.

In light of the patient’s and family’s adamant refusal of limb amputation, the case was reassessed by the multidisciplinary team. The consensus was that in the presence of a patent macrovascular supply, persistent microcirculatory dysfunction was a plausible explanation for the refractory nature of wound healing. Accordingly, on March 7, 2025, a salvage therapeutic strategy targeting microcirculation was initiated, centered on the daily intravenous administration of Sulodexide injection 600 LSU. A clinical turning point was observed on March 23, 2025, with the first appearance of healthy, well-vascularized granulation tissue at the wound base. Objective improvement in microcirculatory perfusion was subsequently confirmed by a follow-up TcPO_2_ measurement of 53.6 mmHg on March 28, 2025. Subsequently, the wound healing trajectory accelerated markedly, and the wound healing trajectory accelerated markedly, and the wound met the criteria for a healed foot ulcer (defined as complete epithelialization without any drainage) on April 26, 2025, a determination made by the attending physician upon clinical examination prior to discharge (8). The patient was ultimately discharged with the limb successfully salvaged.

**Figure 1:**
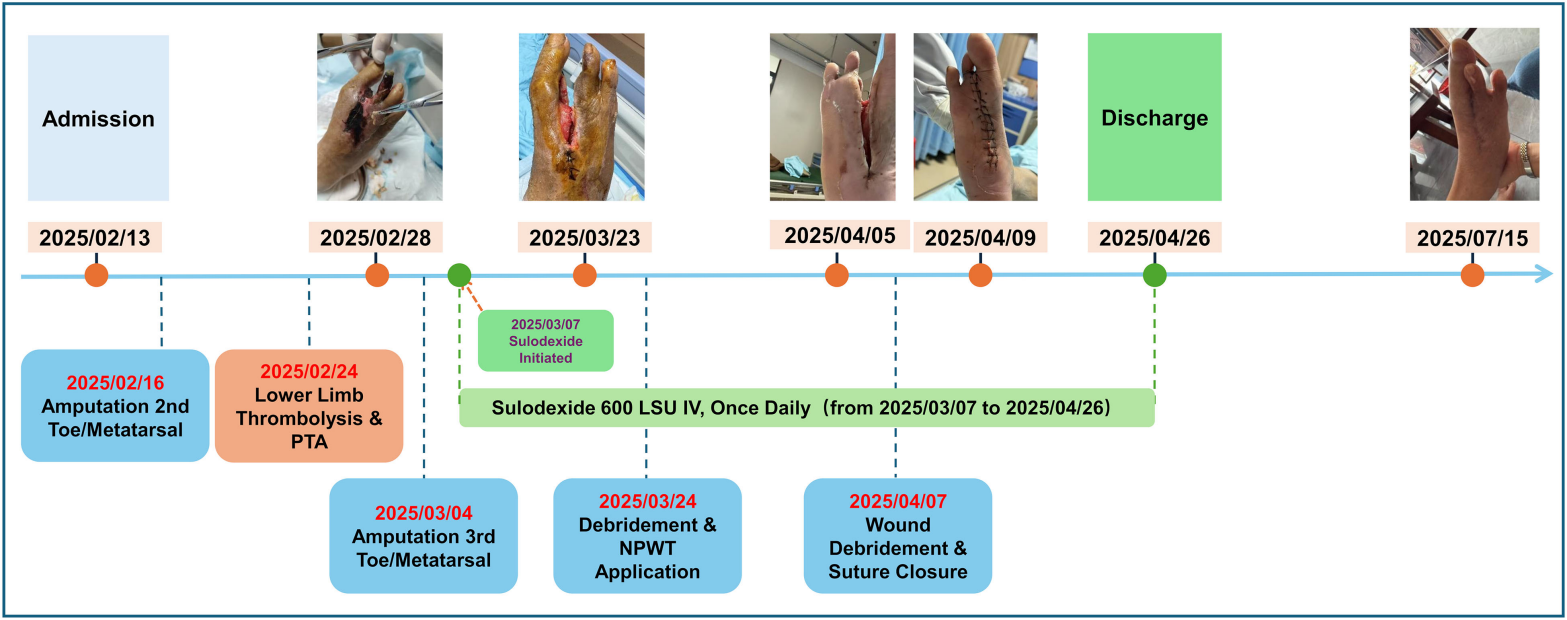
Patient’s treatment timeline and wound evolution.

At a recent follow-up on July 15, 2025, the wound on the right foot was confirmed to have remained healed and stable. There was no evidence of a recurrent foot ulcer, defined as a new ulcer at any site on the ipsilateral foot in a person with a history of ulceration (8). To date, no new ulceration or infectious complications have been observed on the affected limb or elsewhere. A comprehensive timeline of the treatment course and wound evolution is presented in **Figure 1**.

## Discussion

This report documents a case of limb salvage in a patient with severe diabetic foot ulcer. The patient’s management was based on a comprehensive, guideline-adherent framework that included aggressive surgical debridement, infection control, postoperative Negative Pressure Wound Therapy (NPWT, consistent with IWGDF 2023 recommendations), and an angiographically and hemodynamically successful revascularization. The successful outcome was achieved following the initiation of Sulodexide as a salvage therapy. This clinical course suggests that for a specific subset of patients with DFU, targeted intervention aimed at the microcirculation following revascularization may be a critical determinant for achieving wound closure and ultimate limb preservation.

The refractory nature of this patient’s wound can be attributed to severe microcirculatory dysfunction. Although the endovascular angioplasty was technically successful, confirmed by both angiographic patency and a normalized ABI, a stark discordance emerged between this procedural success and the clinical outcome. Specifically, the patient’s rest pain persisted, the affected foot remained cool, and further tissue necrosis developed. This discrepancy was quantitatively supported by TcPO_2_ measurements, which showed negligible improvement after revascularization. This finding strongly indicates an underlying pathology at the microvascular level. Chronic hyperglycemia and its associated metabolic derangements are well-established drivers of widespread endothelial dysfunction (9, 10). Consequently, despite the restoration of macrovascular inflow, perfusion at the capillary level within the wound bed likely remained insufficient. This state of impaired capillary-level perfusion, a form of microcirculatory dysfunction, may have represented a significant pathophysiological barrier to healing in this case (10, 11).

The hypothesis of microcirculatory dysfunction as the primary impediment is strengthened by the exclusion of other major confounders. The patient’s severe infection was resolved, evidenced by normalizing inflammatory markers. Other key variables, including glycemic control, nutrition, edema, and offloading, were also managed according to standard protocols. Despite these measures, the wound continued to deteriorate, isolating impaired tissue perfusion as the most plausible remaining barrier to healing. This clinical scenario presents a distinct therapeutic challenge not fully addressed by the IWGDF 2023 Practical Guidelines, which note that chronic microangiopathy is not typically a primary etiology of DFU non-healing (12). The acute, functional perfusion deficit observed post-revascularization, however, provided a clear rationale for targeting the microcirculation.

Sulodexide’s contribution to the favorable outcome in this case is possibly related to its targeted effects on microcirculatory dysfunction. This interpretation is strengthened by the observation that the clinical turning point occurred only after Sulodexide was added to an otherwise stable therapeutic regimen of intensive inpatient care, which had previously failed to halt wound deterioration. Microcirculatory damage can be caused by ischemia, reperfusion, inflammation, and hypoxia, resulting in endothelial and glycocalyx damage (13).The glycocalyx is responsible for several critical physiologic processes including homeostasis, solute transport, hemostasis, and immunological functions (13). Sulodexide is a glycosaminoglycan mixture composed of 80% low-molecular-weight heparan sulfate and 20% dermatan sulfate, which are natural components of the glycocalyx of vascular endothelial cells (14).Sulodexide’s potential to repair the endothelial glycocalyx may have restored microcirculatory homeostasis within the local wound environment (15). Concurrently, its anticoagulant, antithrombotic, and rheological properties likely contributed to a reduction in microvascular flow resistance (7, 15, 16). The patient reported in this case has peripheral arterial disease. SUAVIS studies have shown that sulodexide can significantly improve pain-free and maximum walking distances in patients with peripheral arterial disease (17). Notably, a prior study reported limited benefit of Sulodexide in a general population of patients with DFU (18). However, the patient cohort in that study was not stratified by their response to revascularization. The present case suggested microcirculatory dysfunction may play an important role in refractory DFU underlying technically successful revascularization, and Sulodexide was a treatment option. Furthermore, throughout the 51-day period of intravenous therapy, this patient experienced no treatment-related hemorrhage or other adverse events, suggesting a favorable safety profile of Sulodexide in this context. Therefore, in this case, Sulodexide may not have merely played an adjunctive therapeutic role but served as a primary driver of wound healing by directly mitigating the underlying microvascular pathology.

The clinical course of this case suggests that for highly selected patients with DFU refractory to optimal standard care, targeting microcirculatory dysfunction could be considered as a potential adjunctive or salvage therapeutic strategy. This is particularly relevant for DFU complicated by severe ischemia, where a comprehensive therapeutic paradigm should address both macro- and micro-circular deficits concurrently. Within such a treatment model, the administration of Sulodexide in parallel with revascularization could be considered for patients in whom an underlying microcirculatory impairment is suspected.

The primary limitation of this study is its nature as a single case report and the consequent absence of a comparator or control group, from which a definitive causal relationship cannot be established. Nevertheless, the clear temporal association between the initiation of Sulodexide and the reversal of the wound’s healing trajectory provides hypothesis-generating evidence. Therefore, prospective clinical studies are warranted to validate the efficacy of targeting microcirculatory dysfunction as a foundational therapeutic strategy for this challenging DFU subpopulation.

## Conclusion

For patients with refractory DFU unresponsive to standard therapies, including successful revascularization, addressing the underlying microvascular pathology may be a critical consideration. This case report suggests that Sulodexide may be a valuable salvage or adjunctive therapeutic option. in this specific clinical context, an observation that is strictly hypothesis-generating. While not a substitute for guideline-recommended care, the potential role of this therapy warrants further investigation in prospective clinical studies to improve outcomes for this high-risk patient population.

## Data Availability

The original contributions presented in the study are included in the article/supplementary material. Further inquiries can be directed to the corresponding author.
